# Novel insights into tumorigenesis revealed by molecular analysis of Lynch syndrome cases with multiple colorectal tumors

**DOI:** 10.3389/fonc.2024.1378392

**Published:** 2024-04-25

**Authors:** Alisa Olkinuora, Satu Mäki-Nevala, Sanjeevi Ukwattage, Ari Ristimäki, Maarit Ahtiainen, Jukka-Pekka Mecklin, Päivi Peltomäki

**Affiliations:** ^1^ Department of Medical and Clinical Genetics, University of Helsinki, Helsinki, Finland; ^2^ Department of Pathology, HUSLAB, HUS Diagnostic Center, Helsinki University Hospital and University of Helsinki, HUS, Helsinki, Finland; ^3^ Applied Tumor Genomics Research Program, Research Programs Unit, Faculty of Medicine, University of Helsinki, Helsinki, Finland; ^4^ Department of Pathology, Wellbeing Services County of Central Finland, Jyväskylä, Finland; ^5^ Department of Education and Science, Nova Hospital, Central Finland Health Care District, Jyväskylä, Finland; ^6^ Faculty of Sports and Health Sciences, University of Jyväskylä, Jyväskylä, Finland; ^7^ HUSLAB Laboratory of Genetics, HUS Diagnostic Center, HUS, Helsinki University Hospital, Helsinki, Finland

**Keywords:** Lynch syndrome, exome sequencing, panel sequencing, multiple adenomas, RNF43

## Abstract

**Background:**

Lynch syndrome (LS) is an autosomal dominant multi-organ cancer syndrome with a high lifetime risk of cancer. The number of cumulative colorectal adenomas in LS does not generally exceed ten, and removal of adenomas via routine screening minimizes the cancer burden. However, abnormal phenotypes may mislead initial diagnosis and subsequently cause suboptimal treatment.

**Aim:**

Currently, there is no standard guide for the care of multiple colorectal adenomas in LS individuals. We aimed to shed insight into the molecular features and reasons for multiplicity of adenomas in LS patients.

**Methods:**

We applied whole exome sequencing on nine adenomas (ten samples) and three assumed primary carcinomas (five samples) of an LS patient developing the tumors during a 21-year follow-up period. We compared the findings to the tumor profiles of two additional LS cases ascertained through colorectal tumor multiplicity, as well as to ten adenomas and 15 carcinomas from 23 unrelated LS patients with no elevated adenoma burden from the same population. As LS associated cancers can arise via several molecular pathways, we also profiled the tumors for CpG Island Methylator Phenotype (CIMP), and LINE-1 methylation.

**Results:**

All tumors were microsatellite unstable (MSI), and MSI was present in several samples derived from normal mucosa as well. Interestingly, frequent frameshift variants in *RNF43* were shared among substantial number of the tumors of our primary case and the tumors of LS cases with multiple tumors but almost absent in our control LS cases. The *RNF43* variants were completely absent in the normal tissue, indicating tumor-associated mutational hotspots. The *RNF43* status correlated with the mutational signature SBS96. Contrary to LS tumors from the reference set with no elevated colorectal tumor burden, the somatic variants occurred significantly more frequently at C>T in the CpG context, irrespective of CIMP or LINE-1 status, potentially indicating other, yet unknown methylation-related mechanisms. There were no signs of somatic mosaicism affecting the MMR genes. Somatic variants in *APC* and *CTNNB1* were unique to each tumor.

**Conclusion:**

Frequent somatic *RNF43* hot spot variants combined with SBS96 signature and increased tendency to DNA methylation may contribute to tumor multiplicity in LS.

## 1 Introduction

LS is a relatively common disorder (affects one in 250-400 individuals) with a high lifetime risk of cancer caused by the deficiency of one of the main four DNA mismatch repair genes: *MLH1*, *MSH2*, *MSH6*, or *PMS2*, or by transcriptional silencing of *MSH2* via 3’ deletion of *EPCAM.* Typically, LS families display autosomal dominant inheritance of colorectal cancer (CRC) (and various extracolonic cancers such as endometrial cancer) with a relatively young age at onset (< 50 years) (1, 2). The Amsterdam criteria, used as an aid in the clinical diagnosis of LS, draws heavily on the family history of CRC, but additionally states the need for the absence of polyps to distinguish LS from familial adenomatous polyposis (FAP) (3, 4). Although the cumulative lifetime adenoma burden of LS patients generally stays below ten, recent data has shown that LS may manifest with an elevated polyp count, and that individuals with pathogenic germline variants in different genes may undergo gene-specific tumorigenesis (5–9), creating difficulties for diagnosis. LS patients with an elevated adenoma count are additionally significantly more likely to have an advanced colorectal neoplasia (8).

The adenoma-carcinoma pathway of CRC is grouped into three distinct subtypes: the chromosomal instability (CIN) pathway, the MSI pathway, and the CIMP pathway. LS-associated tumorigenesis is generally thought to arise via MSI pathway, but recently the possibility of copy-neutral loss of heterozygosity for carriers of *MLH1* germline variants as the cause of cancer has been discussed (10). Of all CRCs, about 10% follow an alternative pathway wherein the adenoma precursor is replaced with a serrated polyp. Although the MSI pathway can sometimes give rise to serrated-type tumors, the CIMP adenoma-carcinoma pathway produces adenomas that resemble MSI carcinomas due to hypermethylation of *MLH1* but lack the hallmark genetic disruption in *APC*; instead, tumors form in association with *BRAF* and *KRAS* mutations driven by an exclusive WNT activating *RNF43* mutation in sporadic CRC (11, 12). Once *RNF43* is inactivated in the serrated lesions, they may advance into mucinous adenocarcinomas with a high likelihood of metastasis. Although frequently observed in sporadic CRC, the mechanisms of the accelerated tumor progression and metastatic spread are poorly understood.

We undertook this investigation to explore the molecular background of colorectal tumor multiplicity in LS, the existing knowledge of which is scarce. We determined the constitutional and somatic molecular profiles of three LS cases with multiple adenomas and compared the findings to a cohort of LS patients with no such colorectal tumor multiplicity. We describe distinct tumor profiles that may be associated with increased colon tumor burden in LS.

## 2 Materials and methods

### 2.1 Patients and samples

**Figure 1:**
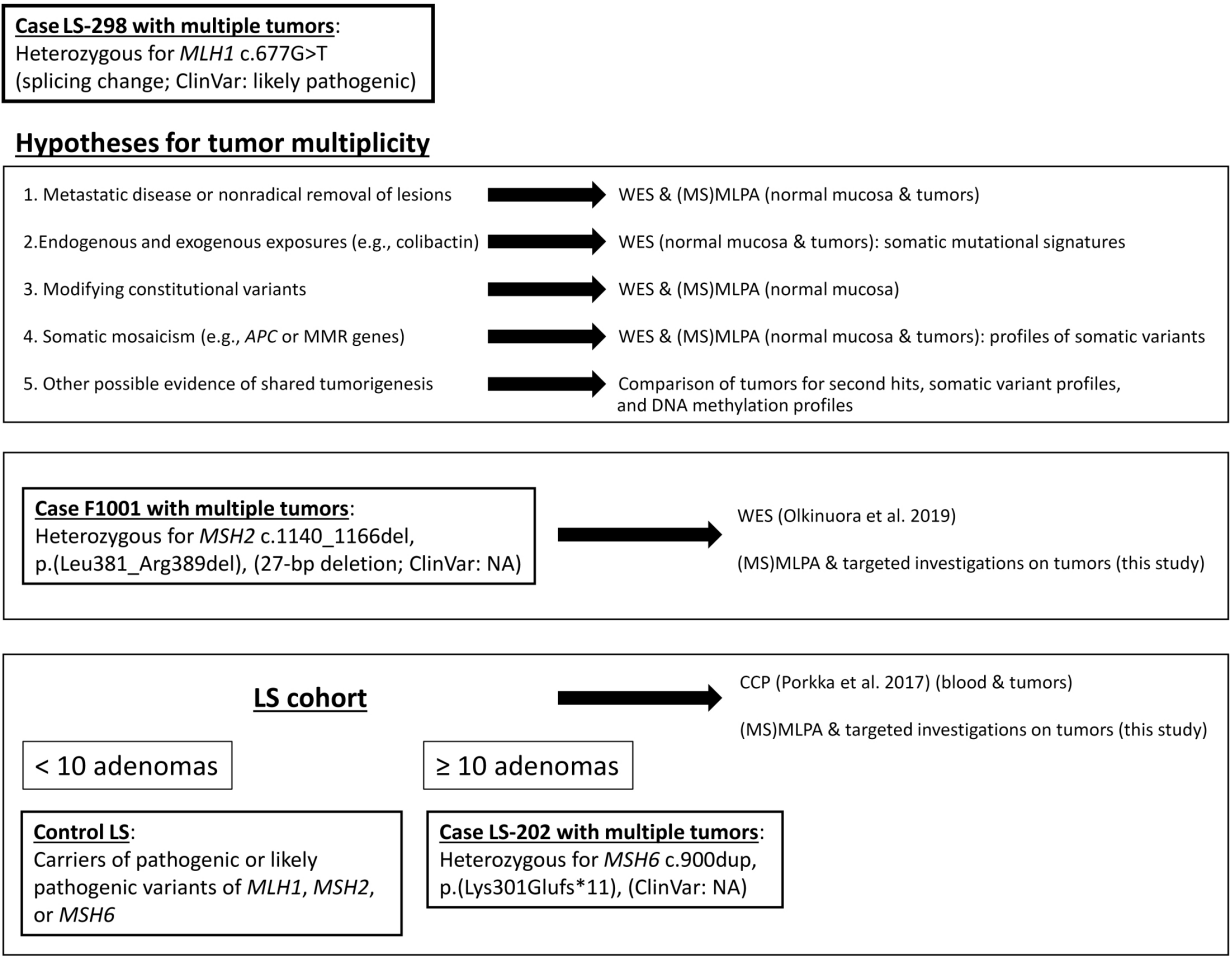
Outline of this investigation. Study cases and cohorts as well as methodological approaches and rationales behind them are shown.

This investigation was carried out on a total of 26 index cases with molecularly confirmed LS (**Figure 1**) from Finland. The patients were ascertained from the Finnish Lynch Syndrome Research Registry. DNA was extracted from blood or archival formalin-fixed paraffin-embedded (FFPE) samples following the protocol presented in Isola et al. (13).

Case LS-298

Our index individual initially presented with a 25 mm mucinous cecal adenocarcinoma and a 40 mm moderately differentiated adenocarcinoma of the ascending colon at 52 years of age. The ascending colon housed a few adenomatous polyps, as well. After right sided hemicolectomy, he developed several tumors in the descending colon as well as renal carcinoma (**Table 1**). Analysis of the germline tissue revealed a pathogenic variant in *MLH1*, c.677G>T, p.(Gln197Argfs*8) (14).

**Table 1:** Molecular features of the case LS-298 in chronological context.

	Carcinomas					Adenomas									
Sample ID	carcinoma 1	carcinoma 2	carcinoma 3	carcinoma 4	carcinoma 5	adenoma 1	adenoma 2	adenoma 3	adenoma 4	adenoma 5	adenoma 6	adenoma 7	adenoma 8	adenoma 9	adenoma 10
	same tumor		same tumor							same tumor					
Years since dx	0	0	0	0	21	12	15	15	15	18	18	21	21	21	21
Tumor information															
Tumor location	ascendens	ascendens	caecum	caecum	transversum	NA	rectosigmoid	rectum	rectum	rectum	rectum	transversum	rectum	rectum	transversum
Histology	NA	NA	Mucinous	Mucinous	Mucinous, signet cell	Villous	Tubular	Tubular	Tubular	Tubuvillous	Tubuvillous	Tubular	Tubular	Tubular	Tubular
Dysplasia						moderate	moderate	high	moderate	moderate	moderate	high	high	high	low
MSI	MSI	MSI	MSI	MSI	MSI	MSI	MSI	MSI	MSI	MSI	MSI	MSI	MSI	MSI	MSI
2nd hit or LOH	No	No	No	No	**Yes**	**Yes**	**Yes**	No	**Yes**	**Yes**	No	**Yes**	No	No	No
TMB (mut/Mb)	34.39	53.03	13.52	22.76	25.39	30.30	39.27	38.55	33.27	32.79	34.64	41.64	42.21	33.79	28.06
Oncogenic event															
APC	–	miss	–	miss	–	trunc	–	–	–	–	–	–	–	trunc	–
CTNNB1	–	–	miss	miss	miss	–	–	–	–	–	–	–	–	miss	–
KRAS	trunc	–	–	–	–	–	–	–	–	–	–	–	–	–	–
TP53	trunc	–	–	trunc	–	–	–	–	–	–	–	trunc	–	–	–
RNF43 hotspot	trunc	trunc	–	–	trunc	–	trunc	trunc	trunc	trunc	trunc	trunc	trunc	–	trunc
MBD4	–	–	–	–	–	–	trunc	trunc	–	–	–	–	–	trunc	trunc
SBS96	No	**Weak**	No	No	**Weak**	**Weak**	**Yes**	**Yes**	**Yes**	**Yes**	**Yes**	**Yes**	**Yes**	**Yes**	**Yes**
CIMP (Weisenberger)	**Yes**	**Yes**	No	NA	No	**Yes**	**Yes**	No	No	No	No	**Yes**	No	**Yes**	No
LINE-1 1m	1.06	0.66	0.75	0.89	0.98	0.76	0.96	0.92	0.95	0.71	0.73	0.95	0.83	0.86	0.78
LINE-1 2m	1.13	0.63	0.73	0.78	0.74	0.82	0.90	0.81	0.89	0.58	0.66	0.94	0.80	0.79	0.79
LINE-1 3m	0.81	0.78	0.79	0.71	0.79	0.73	0.71	0.94	0.90	0.71	0.61	0.73	0.93	0.75	0.91
%C>T at CpG	36.3%	27.9%	14.2%	19.5%	42.2%	48.3%	56.6%	58.3%	56.8%	56.6%	56.8%	52.2%	50.5%	53.8%	54.6%

CIMP was evaluated using the Weisenberger criteria.

trunc, truncating; miss, missense; NA, Not Available.

Case F1001

The index of F1001 had a personal history of several adenomas, leading to the initial suspicion of attenuated familial adenomatous polyposis. He underwent subtotal colectomy at 47 years of age due to the presence of multiple seemingly primary carcinomas with mucinous, and signet cell/mixed histology as well as several adenomatous polyps. Subsequent analysis of the case revealed a 27 bp deletion of *MSH2* c.1140_1166del, p.(Leu381_Arg389del) in the MSH3/MSH6 interaction domain (6). MSI and IHC analyses complied with *MSH2*-associated Lynch syndrome.

Case LS-202

The case LS-202 was initially diagnosed with a moderately differentiated rectum carcinoma and several sigmoid polyps at 57 years of age. He underwent an anterior resection During routine follow-up screens, adenomas were observed at a regular interval, and at the age of 64, he developed two cecal pT2N0 adenocarcinomas alongside few adenomas. Despite being subjected to right-sided hemicolectomy, several adenomas were observed at routine screens. Molecular analysis of germline tissue revealed a likely pathogenic germline alteration in *MSH6*, c.900dup p.(Lys301Glufs*11) (15).

Control LS cohort

Twenty-five LS-tumors (ten adenomas and 15 carcinomas) from 23 LS cases with a typical disease expression were subjected to panel sequencing using the Comprehensive Cancer Panel (CCP) as previously described (15). Somatic mutational data for this cohort is available online by Porkka et al. (15). All control LS patients were confirmed carriers of pathogenic or likely pathogenic germline variants in the MMR genes.

Written informed consent preceded study participation and sample donation. This study was approved by the Institutional Review Boards of the Helsinki University Central Hospital (466/E6/01) and Central Finland Health Care District (10U/2011) approved this study. The National Supervisory Authority for Welfare and Health (Dnro 1272/04/044/07 and Dnro 10741/06.01.03.01/2015) approved the collection of archival specimens.

### 2.2 Exome sequencing and variant prioritization

Exome sequencing (ES) was performed as previously described by Olkinuora et al., or by preparing the library and enriching components with the Twist Core Exome + RefSeq kit and run on Novaseq S1 system at the Institute for Molecular Medicine Finland, FIMM (**Supplementary Table S1**) (6). Sequences were aligned to the human reference genome GRCh37/hg19 using the Burrows-Wheeler Aligner version 0.6.2. Quality control was performed as described by Sulonen et al. (16).

Germline and somatic variant data was annotated using ANNOVAR (17). Variants fulfilling the following selection criteria were selected for further analyses: gnomAD allele frequency < 0.001, nonsynonymous (frameshift, stop gained/lost, missense, disrupting donor/acceptor site variants) and predicted pathogenic with at least five of six programs assessing protein function in silico (for missense changes). DNA methyltransferases and genes with the methyl-CpG binding domain according to the HGNC database (https://www.genenames.org/) were taken for closer inspection.

The clinical enrichment analysis of MAFtools was used to compare germline variant distribution between groups (18). The input variants were restricted to the CCP panel, and all output genes were manually confirmed for sufficient coverage using Integrative Genomics Viewer (IGV).

### 2.3 Somatic variant profiling

VarScan2 variant detection algorithm version 2.3.2 was applied to tumor-normal pairs to identify non-synonymous somatic variants from ES data. SnpEff version 4.0 with the Ensembl v68 annotation database (https://www.ensembl.org) was used to annotate the variant data. Due to the limited number of genes in the CCP used in the original analysis of the control LS dataset, the somatic exome data was filtered to match the sequenced regions of CCP for downstream analyses comparing two cohorts. Variants with a VarScan2-derived somatic p-value less than 0.01 were selected for somatic mutational signature analysis, which was carried out using the R package MutationalPatterns (19). The single-base substitution (SBS) somatic mutation matrix was additionally repaired for FFPE-derived artefacts using the FFPEsig python package (20). The signatures present in the tumors were evaluated by running NMF factorization rank estimation on the mutational matrices with the NMF R package to draw *de novo* signatures with 1000 iterations. The resulting *de novo* signatures were then mapped against the SBS signatures by Degasperi et al. and 18 insertion-and-deletion (ID) signatures recognized by the COSMICv3.1 database (cancer.sanger.ac.uk) (21).

Tumor heterogeneity and driver mutation analyses were carried out using the R package MAFtools (18).

### 2.4 Loss of heterozygosity (LOH)

Possible loss of heterozygosity was evaluated on germline mutation loci based on either the results ES or fragment analyses by comparing the allele or peak ratios of tumor samples to the corresponding ratios in the normal sample by applying the following formula: LOH (or allelic imbalance) ratio (R) = (A/B)T/(A/B)N. LOH ratios 1.67 ≥ R ≤ 0.60 were considered indicative of strict LOH, and R= 0.6 – 0.8 and R= 1.25 – 1.67 putative LOH (22).

### 2.5 Microsatellite instability analysis

Mononucleotide markers BAT25 and BAT26 were used to classify samples MSI or MSS. Samples were considered MSI when at least one marker showed instability. Recurrent frameshift mutations at coding microsatellites identified by the analysis of tumor tissue were confirmed in adjacent normal tissues with the following fluorescent markers: CASP5_fwd, 5’-AACTCTTTAAGCTGTGCCCA-3’; CASP5_rev, 5’-TCTACCAAGATCAGGGCCTT-3’; LTN1_fwd, 5’-GAAGCTGATGTTGAGTCCGT-3’; LTN1_rev, 5’-GCTTTCAAGTATCTCATCAGCA-3’; MARCKS_fwd, 5’-CCGCCTCCTCGACTTCTT-3’, MARCKS_rev, 5’-CCGCTCAGCTTGAAAGACTT-3’; NCAM1_fwd, 5’-TACTCAGCCTGGCAATTGTC-3’; NCAM1_rev, 5’-ATTGTAATCTGCTGGCTGGG-3’; RNPC3_fwd, 5’-GCAAAAGAGCAAGATCGAGT-3’; RNPC3_rev, 5’-ACTTGCTAGTCTGAAAACAA-3’; SLC22A9_fwd, 5’-TGCAGTCAACTCACTTCTCA-3’; SLC22A9_rev, 5’-CGTAAAGGACAGGAGGGAGA-3’; TAF1B_fwd, 5’-CTGCAGAGATATCAGGAAGTTACA-3’; TAF1B_rev, 5’-CATCATGAAGGTGAAAGATGTGA-3’; USP48_fwd, 5’-CTTTAGCAAAGCAAGAAAAGC-3’; USP48_rev, 5’-TGGAAACTCAGGAGCCTTTG-3’; RNF43_Arg117fs_fwd, 5’-TCTGGAGCCTGGATTCATCA-3’; RNF43_Arg117fs_rev, 5’- GCGAAGTGTGAGTCTACCTT-3’; RNF43_Gly659fs_fwd, 5’- CTCTCTGCCCGACACCCA-3’; RNF43_Gly659fs_rev, 5’-TTGCATCCTGGGGCCGAG-3’. Fragment analysis was performed at the Institute for Molecular Medicine Finland FIMM Genomics unit supported by HiLIFE and Biocenter Finland.

### 2.6 Germline and somatic methylation analyses

Possible presence of CIMP was evaluated by methylation-specific multiplex ligation-dependent probe amplification (MS-MLPA) using SALSA MS-MLPA probemix ME042-C2 (MRC Holland, Amsterdam, the Netherlands) as previously described (23). CIMP+ status was given when three out of five genes exhibited dosage ratios above a threshold level for hypermethylation as defined by the Weisenberger panel (24). Thresholds for hypermethylation were calculated as described previously (23).

The methylation status of LINE-1 was determined using custom MS-MLPA probes described by Pavicic et al. using commercially available reagents from MRC Holland (25).

### 2.7 Genomic rearrangements in MMR genes

Blood or normal mucosa FFPE -derived DNA of the study cases were examined for large genomic rearrangements using multiplex ligation-dependent probe amplification (MLPA) according to manufacturer’s (MRC-Holland, Amsterdam, the Netherlands) instructions. SALSA MLPA P003-D1 and SALSA MLPA P072-D1 were used for MLH1/MSH2 and MSH6/MUTYH, respectively, whereas PMS2 was investigated by SALSA MLPA P008-C1. The results from fragment analysis were analyzed by Coffalyser™ (MRC-Holland, Amsterdam, the Netherlands).

### 2.8 Immunohistochemistry for MMR protein expression

Archival FFPE tissue specimens from index cases were stained using anti-MLH1 (clone G168-15; Pharmingen), anti-MSH2 (clone FE-11; Calmiochem/Oncogene Research), and anti-MSH6 (clone 44; Transduction Laboratories) antibodies according to Thiel et al. (26). Dako Envision+ System, DAB Peroxidase was applied according to manufacturer’s instructions for visualization.

### 2.9 Statistical analysis

Analysis of statistical significance between groups were carried out by Fisher’s Exact or two-tailed ANOVA for normally distributed data, and by Kruskal-Wallis analysis for non-parametric data. Tukey HSD or Dunn’s test was used for *post hoc* analyses for ANOVA and Kruskal-Wallis, respectively. All analyses were conducted on R v.4.2.3.

## 3 Results

We sought to discriminate whether the underlying reason for the multiplicity of colorectal tumors in case LS-298 and two other similar cases (**Figure 1**) was due to 1) metastatic disease or nonradical removal of initial lesions; 2) mutagenic stressors, e.g. colibactin; 3) modifying germline variants resulting in ultrahypermutability; or 4) somatic mosaicism, e.g. potential constitutional mismatch repair deficiency syndrome (CMMRD) or involvement of *APC* that might have escaped detection.

### 3.1 Somatic mutation profiling of tumors

All colorectal tumors from LS-298 were MSI although somatic mutation or loss of the wild type allele of *MLH1* was detectable in only 7 tumors (58.3%). The tumors investigated from F1001, LS-202, and the control LS cohort were likewise microsatellite unstable.

To rule out metastatic disease, we compared somatic variants identified by our tumoral analyses (**Supplementary Table S2**) in all available samples. While the number of shared variants occurred at the same rate in the tumors of LS-298 as in the available controls, by ES analyses, we observed several frameshift-type variants shared across tumor samples of our index case. The rate of shared frameshift variants was not significantly different from the unrelated control LS tumors, however, which indicated that the frameshift variants likely represented mutational hotspots typical of LS. As the number of shared missense and synonymous somatic variants was low and occurred at a similar rate as the control group, the likelihood of metastatic disease or shared clonal origin was low.

When considering driver genes for colorectal neoplasia recognized by the COSMIC database (cancer.sanger.ac.uk/cosmic), most frameshift variants were shared at equal overall proportions in our sample cohorts. Although control LS adenomas acquired variants in *APC* at a higher rate than the tumors of LS-298 (48% (12/25) versus 26.67% (4/15), respectively), the difference was not statistically significant. Two of the three (66.7%) tumors from LS-202 also harbored somatic variants in *APC* (**Supplementary Figure S1B**). Interestingly, the adenomas and carcinomas of LS-298 frequently showed frameshift variants in *RNF43* with high variant allele frequency (median VAF= 44.4; range = 15.69-62.3; **Supplementary Table S2**). As these variants mostly targeted the recognized mutational hotspots of the gene, G659 (G_7_ repeat) and R117 (C_6_ repeat), we screened the control LS datasets for the hotspot mutations as the CCP panel does not cover the *RNF43* gene (27). Frameshift mutations in *RNF43* hotspots occurred significantly more frequently in our study case and LS individuals with multiple tumors (*p* = 0.0001 by Fisher’s Exact Test; **Supplementary Table S3**); 73.3% (11/15) tumor samples of LS-298, and 60.0% (9/15) of F1001 tumors, versus 10% (2/15) of tumors from control LS with sufficient DNA for testing. The single tumor of LS-202 with enough DNA for testing did not carry any *RNF43* hotspot mutations.

Due to the high VAFs of several frameshift variants in coding microsatellite regions of genes in our initial analyses (**Supplementary Table S2**), we analyzed several normal mucosa samples for the possibility of MSI in non-malignant tissue. Although we failed to capture MSI using the typical BAT25/26 markers in most of the normal mucosa samples, we observed instability at selected markers (**Supplementary Table S4**), which could indicate “field defects” of hypermutated hotspot loci, previously reported to occur healthy mucosa of patients with sporadic CRC (28). No *RNF43* hotspot variants were observed in normal tissue, suggesting later involvement in tumorigenesis despite the very high VAF in tumor tissue. Although our normal mucosa samples were evaluated by a pathologist, we cannot eliminate the possibility of tumor contamination or presence of individual cells with neoplastic potential within the sample.

**Figure 2:**
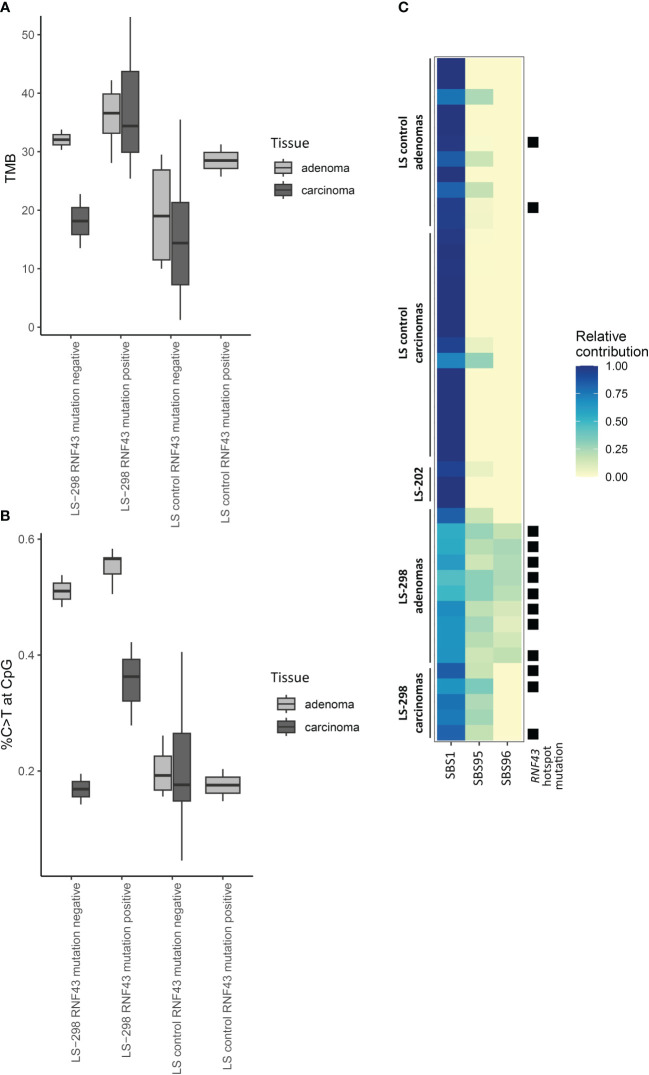
Effect of *RNF43* mutation status on molecular tumor features of LS-298 vs. control LS cohort. Tumor mutational burden **(A)**, percentage of C to T transitions at CpG sites **(B)**, and mutational signatures **(C)** are illustrated. *RNF43* hotspot mutation status is indicated with a solid square.

Typical of LS, majority of the tumors were hypermutated (with 10 somatic variants/Mb as a cut-off) and the rate of somatic variants at C>T context was elevated for adenomas and carcinomas for both LS-298 and control LS dataset (**Supplementary Figure S1A**; **Supplementary Table S2** (15);). The adenomas of LS-298 displayed higher average tumor mutational burden (TMB) than control LS adenomas or carcinomas (*p* = 0.01748 and *p* = 0.00093, respectively, by Mann-Whitney U-test), while the carcinomas of LS-298 did not significantly differ from control LS adenomas or carcinomas. However, when mapping the somatic C>T variants in context of CpG regions, we noticed an elevated rate of C>T variants at CpGs in the tumors of LS-298, being particularly pronounced in the adenomas (*p* = 0.0025 and *p* = 0.000031 vs control LS adenomas and carcinomas, respectively, by Wilcoxon pairwise test). When comparing TMB and the %C>T at CpG of samples with *RNF43* hotspot mutations to those without, we noticed an increase in the mutational load and the percentage of variants occurring at CpG sites (**Figures 2A, B**). While the differences are notable, we were unable to attain statistical significance due to the small number of samples.

Mutational Signature analyses refitted to the consensus signatures (COSMIC v3.1., cancer.sanger.ac.uk) showed typical MSI-associated signatures in all groups including a very strong relative contribution of SBS1 (**Supplementary Figure S2**).

### 3.2 Analysis of sample methylation status

**Figure 3:**
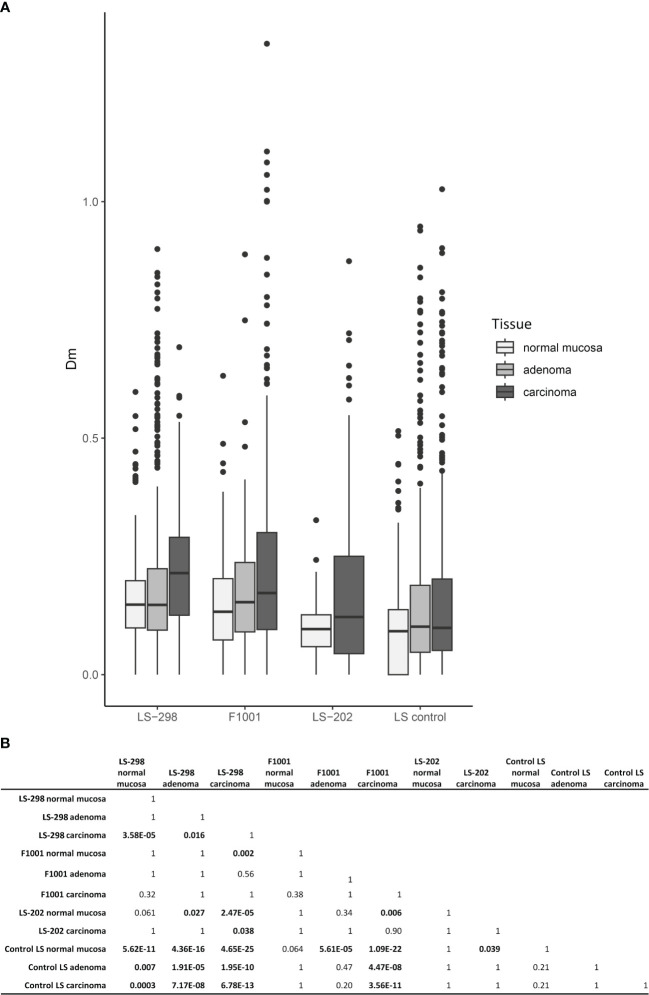
Relationship between CIMP status and tumor multiplicity. Colorectal tumors from LS patients with multiple tumors (LS-289, F1001, and ME16) are compared to those without (LS control group). **(A)** Box plots of distributions of DNA methylation (Dm) values at CIMP marker loci. **(B)** Pairwise Wilcoxon analysis of Dm differences for statistical significance between the sample sets. Statistical significance is indicated with bolded text. P-values were adjusted for multiple testing by Bonferroni correction.

Among the 58 tumor samples where testing was possible, CIMP was observed at a high rate: 50% (29/58) tumors were CIMP positive according to the Weisenberger criteria (24). CIMP was more frequent in carcinomas than in adenomas (*p* = 0.03), and particularly pronounced in the case F1001 (75%, 12/16 of samples were CIMP positive) and LS-202 (100%, 3/3 tumors were CIMP positive). Tumors of LS-298 did not differ significantly from the control LS cases (**Table 1**). However, the overall Dm ratios of the genes covered by the MS-MLPA kit used were higher in all tissue types of LS-298 compared to the matching tissue type of the control LS cases. The Dm ratios of F1001 were comparable to those of LS-298, whereas LS-202 did not differ significantly from tissue-matching control LS group. (**Figure 3**). *RNF43* hotspot-mutation positive samples had no significant correlation to CIMP status contrary to previous studies (29).

Comparison of the genes included in the CCP panel did not reveal a difference between our study cases and controls in their somatic mutational load in genes with a primary function in epigenetic regulation (data not shown). However, prompted by the propensity of somatic variants to occur at the CpG regions, frequent CIMP positivity, and the predominance of SBS1 in our study case’s tumors when comparing the mutational data to COSMIC consensus signatures (**Table 1**; **Supplementary Figure S2**), we compared sample signatures to a specific subset of hypermutator signatures associated with MSI and spontaneous demethylation of C>T to see whether the tumor phenotypes were indicative of *MBD4*-associated tumorigenesis as described by Degasperi et al. (21). The subset signature analysis revealed that majority of tumors of LS-298 harbored the SBS96 signature (**Figure 2C**), whereas tumors from control LS cases unanimously harbored only SBS1 or SBS1 and SBS95. Interestingly, mapping the presence of *RNF43* hotspot mutations to the relative contribution of signatures showed a significant association with a moderate positive correlation with the hotspot mutation status and the strength of SBS96 (*r* = 0.64, *p* = 0.00003897 by Point-Biserial correlation).

Long interspersed elements (LINEs) constitute roughly 17% of the human genome, and typical to retrotransposons, are normally heavily methylated. In cancer, LINEs may activate, inducing downstream genes and/or lead to CIN and in LS, hypomethylated LINE-1s have been shown in association with early-onset CRC as well as an elevated TMB (30–32). Overall, LINE-1 sequences retained their methylated state, and the level of methylation did not correlate with CIMP status (**Table 1**; **Supplementary Table S5**).

### 3.3 Germline features

Our recent discovery of a LS patient with multiple adenomas possibly due to a synergistic effect of the Lynch-associated germline alteration in *PMS2* and a heterozygous germline variant in *NEIL1* also observed in unexplained polyposis families inspired us to look for modifying germline variants in DNA repair genes (33). Our analysis pinpointed *OGG1* VUSes in two individuals: case LS-298 was compound heterozygous for two *OGG1* missense variants, p.Ala330Val and p.Asn331Ser, and case F1001 with several primary carcinomas and adenomas was heterozygous for the *OGG1* missense variant p.Leu259Phe. These variants are very rare in the average population: the missense variants found in LS-298 have a MAF < 0.001 (gnomAD2.1.1) whereas the missense variant observed in the case F1001 is absent in available databases. The clinical significance of these *OGG1* variants remains unknown. There were no *OGG1* germline variants in LS-202.

Inspired by the somatic mutational profile of LS-298 which indicated the involvement of methylation-related mechanisms at play, we analyzed the ES data of this case in more detail for modifying variants involved in the maintenance of methylome. No germline variants of potential clinical significance were detected. (**Supplementary Table S6**). Despite *in silico* enrichment analyses and thorough manual analysis of ES and (MS-)MLPA data, no potentially pathogenic germline variants were detected in the established CRC-associated susceptibility genes, excluding the possibility of CMMRD or involvement of other high-penetrance CRC genes (1). Our *MLH1* and *CTNNB1* analyses did not suggest the involvement of copy-neutral LOH in tumors (data not shown).

## 4 Discussion

Elevated adenoma burden has been shown in about 6% of LS patients, and multiple cumulative adenomas are particularly observed in carriers of pathogenic *MSH6* and *PMS2* gene variants as well as individuals with CMMRD (8, 34, 35). Understanding the history of adenomas of LS patients is important as those with > 10 cumulative adenomas are much likelier to develop advanced neoplasia (8, 34). Familial risk for tumor multiplicity has been proposed before and some polygenic factors have been identified (36). While our analyses did not reveal promising germline alterations predisposing one to an elevated tumor load, the fact that two of the three LS cases with multiple tumors shared the same hallmark molecular features suggests an intrinsic susceptibility to tumor multiplicity.

Schemes classifying the development of CRC focus mainly on the intrinsic features of tumor cells, e.g. histopathology and molecular characteristics. While these traits are generally thought to be divergent, some molecular characteristics overlap in tumors with differing histology (37). As the molecular and histopathological features of a tumor are of critical prognostic and predictive significance, accurate classification of subtypes of CRC is crucial. The classic adenoma-carcinoma sequence thought to occur in LS-associated tumorigenesis has been challenged since recent reports have indicated that carriers of pathogenic *MLH1* variants may undergo the so-called copy-neutral LOH which results in the simultaneous loss of *CTNNB1* and *MLH1* due to genomic rearrangement. This would result in a short or adenoma-free progression to cancer (10). Additionally, while the occurrence of serrated adenomas in individuals with LS is thought to be comparable to the general population, certain molecular features typical of serrated pathway in colorectal cancer are observed in LS as well; namely the frequent occurrence of MSI and CIMP (15, 38, 39).

The MMR system is integrally involved in the maintenance of DNA methylation, and it has been proposed that MMR uses hemimethylated DNA as a strand-discriminator when targeting the newly synthesized DNA (40). This tight link with defective MMR system and changes in genomic methylation is often seen in LS; LS-associated adenomas and carcinomas acquire hypermethylation of select genes (41), and sometimes CIMP (15, 30). In the sporadic setting, the presence of CIMP has been shown to associate with worse prognosis irrespective of MSI, as well as an increased risk of developing metachronous advanced colorectal lesions (42, 43). Interestingly, Murcia et al. note that a positive CIMP status also predicted the formation of serrated polyps in unselected group of CRC patients (43). Conversely, studies of sporadic CRC cases revealed an association between frequent metastases at diagnosis and the absence of CIMP (44). While a large proportion of tumors from our study cases were CIMP positive, the number was not significantly higher compared to the control LS dataset from the same population. Although we could not assess the genome-wide methylation status of our study cases, using LINE-1 MS-MLPA as a proxy for genome-wide methylation status, we noted variable methylation dosages that did not correlate with CIMP or TMB (**Supplementary Table S5**). While LINE-1 hypomethylation has been associated with poorer prognosis in individuals with LS, there was no consistent hypomethylator effect in tumors from LS-298 (45). On the contrary, several tumors showed high levels of methylation at LINE-1 loci (**Supplementary Table S5**), possibly indicating genome wide hypermethylation as was suggested by our CIMP analyses (**Figure 3**).

Using C>T at CpG islands as a proxy for mutagenesis occurring at methylated cytosines further indicated that the tumors of case LS-298 were largely hypermethylated. C>T at CpG is thought to arise due to spontaneous deamination of 5-methylcytosine, and elevated rate of C>T at CpG may therefore reflect increased mutational processes resulting in deamination, increased occurrence of methylated CpGs, or a combination of the two (46). In addition to defects in DNA repair, spontaneous deamination can occur due to mutagenic exposure, by for example, *Helicobacter pylori* has been suggested to trigger AID, which, in turn, has been thought to lead to gastric cancer (47–50). The *H. pylori* induced pathology also seems to be aggravated by somatic variants in *RNF43* (51). Our mutational analyses did not show any association to SBS2 (APOBEC/AID deaminases), SBS9 (AID) or SBS24 (Aflatoxin), indicating a different mutagenic origin of the observed phenotype. Moreover, we found no discernible SBS88 signature that is linked to colibactin and was reported to occur in colorectal tumors from *NTHL1*- and *MUTYH*-associated polyposis patients (52). The molecular features of tumors that were available for testing in our case F1001 mimicked those of LS-298, allowing us to hypothesize that similar tumorigenic mechanisms play a role in both individuals.

*RNF43* is a tumor suppressor negatively regulating the Wnt signaling pathway by degrading the Wnt Frizzled-LRP5/6 complex. This prohibits the phosphorylation of β-catenin and allows activation of downstream target genes (53). Somatic *RNF43* variants typically show mutual exclusivity with somatic *APC* variants, as was evident in our patients (**Supplementary Figure S1B**), probably owing to the genes’ importance in β-catenin destruction (27, 54, 55). As *APC* is integrally involved in the development of adenomas by both germline and somatic inactivation, *RNF43* defects could promote the formation of adenomas (56). The hotspot somatic variants, G659 (G_7_ repeat) and R117 (C_6_ repeat), occur at short coding microsatellites, targets for MSI-induced somatic mutations. While the hotspot variant at R117 has proven to reduce the functionality of *RNF43*, the somatic hotspot variant at G659 might activate PI3K/AKT signaling instead of affecting the Wnt pathway directly (57–59). In our patient cohort, the hotspot variant at G659 was more prevalent though the variant at R117 was frequent as well (**Supplementary Table S3**). The PI3K/AKT pathway regulates DNA methylation via phosphorylation of AKT, which might explain the hypermethylated phenotype present in our tumor samples with *RNF43* hotspot variants (60). The involvement of PI3K/AKT pathway might also contribute to tumor multiplicity: germline variants in *PTEN*, a regulator of PI3K/AKT, confer a hamartomatous tumor syndrome, characterized by multiple hamartomatous polyps in the colorectum (61). Interestingly, Fang et al. noted PI3K inhibitors selectively targeted cells with the G659 hotspot variant, suggesting the importance of PI3K inhibition as a therapeutic option for individuals with the G659 hotspot variant (57).

Somatic *RNF43* variants in sporadic CRC are frequently observed and show strong correlation with MSI (27, 62). They have also been reported in strong association with the *BRAF* V600E somatic mutation as well as an aggressive tumor biology, and sporadic CRC patients with both mutations seem to benefit from a specific therapy regimen (43, 54, 63, 64). Though *BRAF* V600E has a very tight association with the CIMP tumor phenotype in sporadic CRC, LS tumors very rarely harbor the *BRAF* V600E driver mutation, to the extent that the presence of *BRAF* V600E can be used as a marker predicting the absence of LS (65). It is therefore perhaps perplexing that somatic variants in *RNF43* are less frequently reported in association with CIMP positivity, and analyses of sporadic serrated polyps have failed to find this enrichment altogether (29, 66). Perhaps, then, somatic variants in *RNF43*, while associated with *BRAF* V600E, are not part of the cascade of events that result in CIMP, but instead in another, yet uncharacterized tumorigenic mechanism that is governed by changes in the methylome. This is supported by our observation that while MSI-associated signatures were strongly present in our LS cases, available tumors from LS-298 with *RNF43* hotspot variants had a significant association to SBS96 (**Figure 2**) which is suggested to arise from aberrant maintenance of DNA methylation due to pathogenic germline *MBD4* variants (67).

Germline variants in *RNF43* have been proposed to cause a serrated polyposis phenotype and somatic mutations in the gene have been suggested to take part in the formation of sporadic serrated polyps (27, 68). While pathologist’s reports of the tumors with high allele frequency *RNF43* variants showed no serrated histology, several molecular features align with the so-called serrated pathway to CRC as discussed above but lacked the characteristic hypermethylation of *MLH1*. Instead, carcinomas of LS-298 frequently represented mucinous subtype with occasional signet cells. Although several features of carcinomas and adenomas of our cases with multiple tumors were common with our index case LS-298, incomplete medical records prohibit us from drawing firm conclusions about possible association between this histology and observed mutational features in our sample sets. Mucinous carcinomas are a rare histological subtype that have clear molecular and clinicopathological features compared to non-mucinous carcinomas. *RNF43* is frequently mutated in mucinous ovarian and pancreatic carcinomas, and mucinous differentiation is often found in serrated colorectal carcinomas, to which germline *RNF43* variants are thought to predispose (68–72). Furthermore, analyses of inflammatory bowel disease (IBD)-associated CRCs have noted an association between chronic inflammation and somatic *RNF43* variants and mucinous or signet-ring cell histological subtypes (73). Interestingly, Fujita et al. noted a tendency of IBD-associated carcinomas to harbor C>T mutations at CpG context, which correlated with the observed methylation levels at CpG sites (73). LS-associated tumorigenesis has a strong immunogenic component, and although outside the scope of this manuscript, we hope future research will shed light on the immunogenic markers of LS with a severe tumor burden (74).

In summary, although our germline analyses did not reveal a potential explanation for tumor multiplicity in our LS individuals, we describe a non-random association of *RNF43* hotspot variants to hypermethylator tumor phenotype that is distinct from the classical *BRAF* V600E-associated CIMP. The *RNF43* variants were likely the result of MSI that preceded the loss of the wild-type MMR allele as MSI was observed at select markers in normal tissue before the loss of wild type MMR gene allele and LOH and 2^nd^ hits were variably observed in the tumors. No shared molecular features of tumors additionally indicate that somatic events arose independently, indicating an increased incidence of mutagenesis. Analyses of larger multinational patient cohorts are needed to fully understand the association of serrated tumor pathway and multiplicity of colorectal tumors in LS. As our sequencing data is based on exome and panel sequencing, we may miss important modifying factors in genes or intergenic regions not covered by the sequencing panel.

## Data availability statement

The datasets for this article are not publicly available due to concerns regarding participant/patient anonymity. Requests to access the datasets should be directed to the corresponding author.

## Ethics statement

The studies involving humans were approved by Institutional Review Boards of the Helsinki University Central Hospital (466/E6/01), Central Finland Health Care District (10U/2011) and National Supervisory Authority for Welfare and Health (Dnro 1272/04/044/07 and Dnro 10741/06.01.03.01/2015). The studies were conducted in accordance with the local legislation and institutional requirements. The participants provided their written informed consent to participate in this study. Written informed consent was obtained from the individual(s) for the publication of any potentially identifiable images or data included in this article.

## Author contributions

AO: Conceptualization, Data curation, Formal analysis, Investigation, Methodology, Software, Validation, Visualization, Writing – original draft, Writing – review & editing. SMN: Conceptualization, Formal analysis, Investigation, Writing – original draft, Writing – review & editing. SU: Investigation, Writing – original draft, Writing – review & editing. AR: Investigation, Project administration, Writing – original draft, Writing – review & editing. MA: Writing – original draft, Writing – review & editing, Conceptualization, Data curation, Resources. JPM: Funding acquisition, Investigation, Writing – original draft, Writing – review & editing, Project administration. PP: Conceptualization, Funding acquisition, Investigation, Supervision, Validation, Writing – original draft, Writing – review & editing.

## Supplementary material

Supplementary Figure 1 Molecular comparison of case LS-298 and LS-202 to the control LS cohort. **(A)** Oncoplot depicting the 20 most commonly mutated genes. Sample-wise TMB is indicated by the barplot on top. **(B)** Distribution of APC mutations in LS-298 versus control LS. The analysis was restricted to genes present in the smaller CCP panel.

Supplementary Figure 2 Relative contribution of sample signatures mapped against COSMICv3 consensus signatures. All samples were repaired for FFPE-derived artefacts by FFPEsig.
