# Supplementary figures and images for: Novel insights into tumorigenesis revealed by molecular analysis of Lynch syndrome cases with multiple colorectal tumors
Source: Front Oncol. 2024 Apr 25;14:1378392. doi: 10.3389/fonc.2024.1378392 (PMC11079657; doi:10.3389/fonc.2024.1378392)

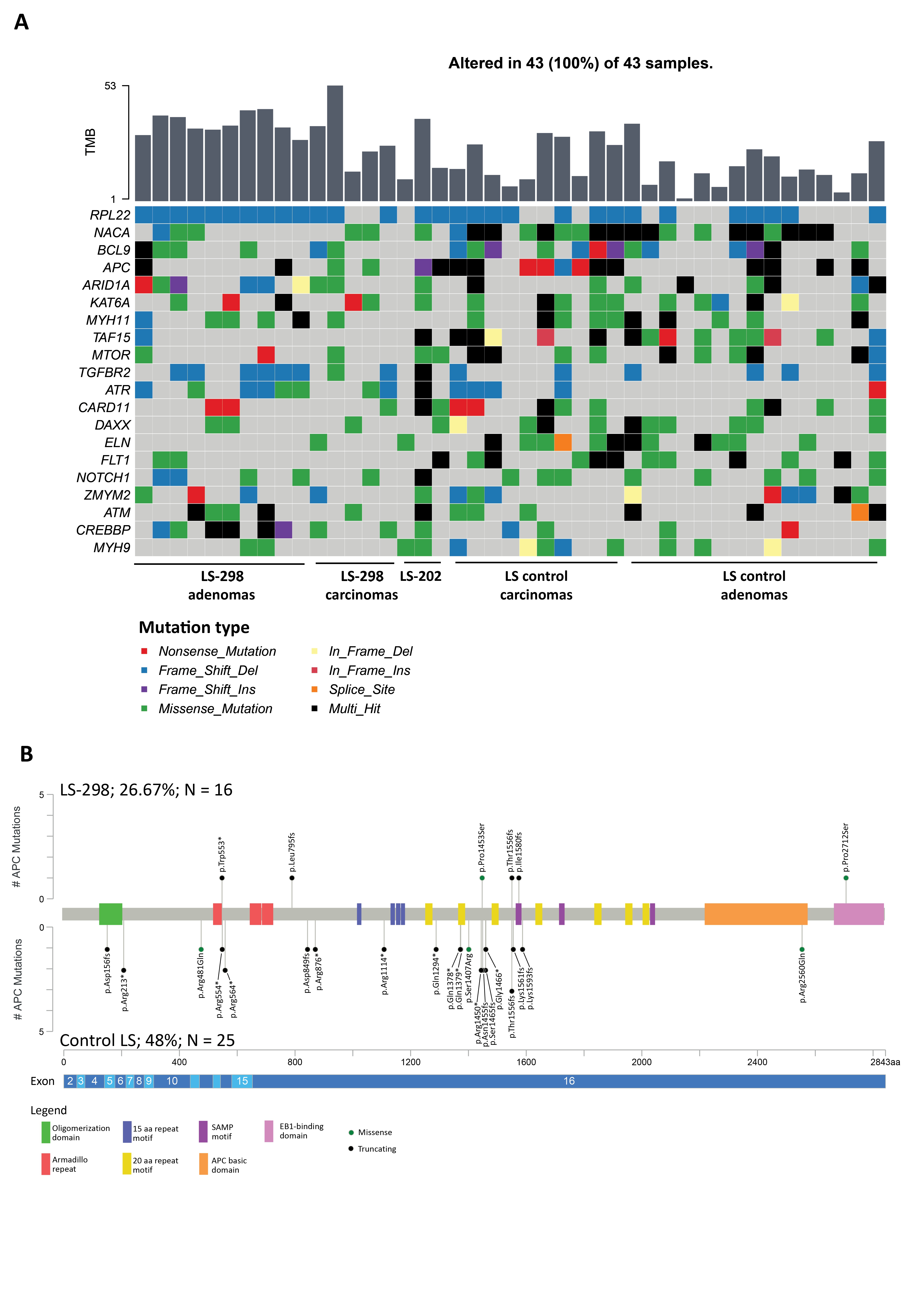

Supplement: Supplementary Figure 1 — Molecular comparison of case LS-298 and LS-202 to the control LS cohort. (A) Oncoplot depicting the 20 most commonly mutated genes. Sample-wise TMB is indicated by the barplot on top. (B) Distribution of APC mutations in LS-298 versus control LS. The analysis was restricted to genes present in the smaller CCP panel. [file Image_1.jpeg]

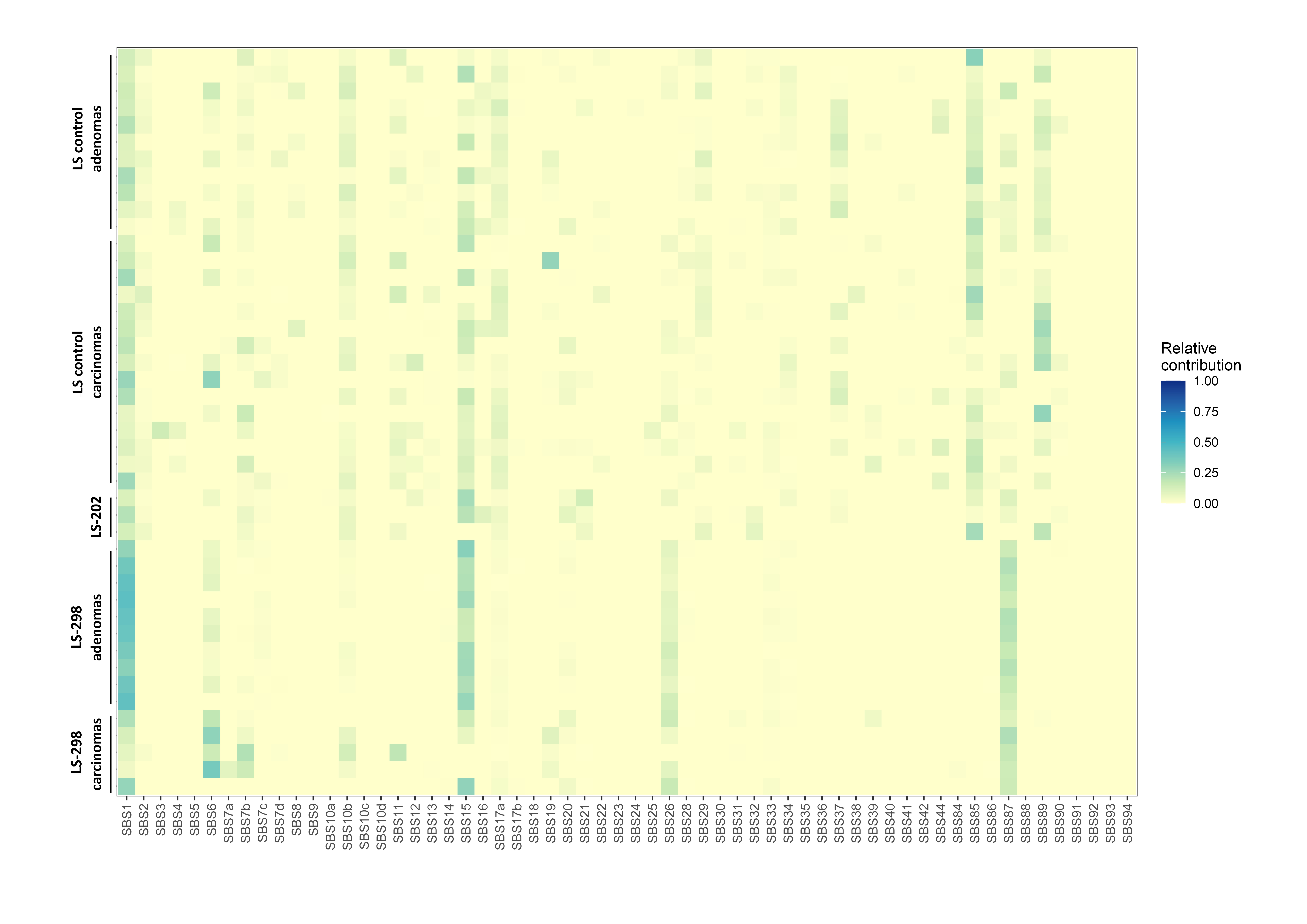

Supplement: Supplementary Figure 2 — Relative contribution of sample signatures mapped against COSMICv3 consensus signatures. All samples were repaired for FFPE-derived artefacts by FFPEsig. [file Image_2.jpeg]
